# hsa_circ_0002980 prevents proliferation, migration, invasion, and epithelial-mesenchymal transition of liver cancer cells through microRNA-1303/cell adhesion molecule 2 axis

**DOI:** 10.18632/aging.205317

**Published:** 2023-12-19

**Authors:** Yu Wang, Zhenlin Li, Jun He, Wenxiang Chen, Yiming Li, Xiangmei Chen, Junjie Liang, Qiangfeng Yu, Jianyin Zhou

**Affiliations:** 1 Department of Hepatobiliary and Pancreatic Surgery, Zhongshan Hospital, Xiamen University, Xiamen, Fujian 361004, China; 2 Department of Surgical Clinical, HeZe Medical College, Heze City 274022, China; 3 The Second Department of General Surgery, Zhuhai People’s Hospital, Zhuhai, Guangdong 51900, China

**Keywords:** hsa_circ_0002980, liver cancer, microRNA-1303, cell adhesion molecule (CADM) 2

## Abstract

Background: Liver cancer (LC) is a rare malignancy. Circular RNA (circRNA) dysregulation is associated with LC metastasis. hsa_circ_0002980 was found to be unexpectedly downregulated in LC tissues; however, its specific function remains unclear.

Methods: hsa_circ_0002980 expression was confirmed using RT-qPCR. The effects of circ_0002980 on the proliferation, metastasis, and EMT-related proteins of LC cells were assessed using clone formation, flow cytometry, Transwell assays, and Western blotting. The relationship between circ_0002980 and miR-1303 or miR-1303 and CADM2 was analyzed using a dual-luciferase reporter assay. Thereafter, the influence of these three genes on LC cell progression was determined through rescue experiments.

Results: hsa_circ_0002980 expression was lower in LC. circ_0002980 overexpression inhibited the proliferation, migration, invasion, and EMT of LC cells. In addition, circ_0002980 specifically binds to miR-1303, and the accelerated effect of miR-1303 overexpression on LC progression was partially reversed by circ_0002980. Moreover, miR-1303 can also target CADM2, and CADM2-mediated prevention can also be attenuated by miR-1303 overexpression.

Conclusions: In LC cells, circ_0002980 upregulation prevents cell proliferation, metastasis, and EMT by affecting the miR-1303/CADM2 axis. Therefore, this axis may be a novel therapeutic target in LC.

## INTRODUCTION

Liver cancer (LC) is a rare primary malignant tumor [1]. Worldwide, it ranks fourth in terms of lethality and sixth in terms of incidence among all fatal oncological diseases [2]. High metastasis and invasiveness are the key factors that cause the high recurrence of LC [3]. Of the patients with LC, 70% of those who are diagnosed early and undergo hepatectomy or liver transplantation achieve a 5-year survival [4]. However, owing to the lack of obvious symptoms in the early stages, 80% of patients are in the progressive stage of disease at first diagnosis [5]. Surgical resection is not possible in patients with advanced LC, resulting in poor prognosis [6]. Currently, early screening for LC mainly relies on abdominal ultrasonography and serum alpha-fetoprotein (AFP) testing, which have relatively limited sensitivity, especially in patients without cirrhosis [7]. Therefore, it is crucial to clarify the mechanisms underlying LC development and identify new specific biomarkers and therapeutic targets.

Circular RNA (circRNAs) is a newly discovered class of endogenous non-coding RNA molecules [8]. circRNAs are structurally conserved, stable, abundantly expressed, and tissue- and cell-type specific [9]. These characteristics make circRNAs potential diagnostic markers for diseases [10]. Additionally, circRNAs have gene regulatory potential and can regulate multiple cell biological processes including proliferation, invasion, autophagy, apoptosis, and metabolism, etc., [11]. The aberrant expression of circRNAs has been associated with disease prognosis [12]. With the development of high-throughput sequencing, a growing number of circRNAs, which may serve as biological markers, have been mined for aberrant expression in LC [13]. Recent research has certified that circRNA is critical in LC progression [14, 15]. In our study, we discovered that hsa_circ_0002980 was weakly expressed in LC tissues, suggesting that it could be used as a diagnostic marker for patients with LC. However, there have been no functional or mechanistic studies on hsa_circ_0002980 in LC. This is the first study to explore the effects and potential mechanisms of action of hsa_circ_0002980 in the proliferation and metastasis of LC cells.

Cell adhesion molecules (CADM) comprise a family of proteins that maintain cell polarity [16]. Most CADMs belong to the immunoglobulin superfamily, which plays a key role in biological processes such as differentiation, development, proliferation, and metastasis of tumor cells [17, 18]. Among these, CADM2, an obesity-related gene, is involved in cellular energy metabolic processes [19]. Recent studies have shown that CADM2 may serve as a cancer-suppressing gene that prevents cell proliferation and metastasis [20–22]. For instance, CADM2 inhibits breast cancer EMT and metastasis [20]. Additionally, the upregulation of CADM2 promotes glioma cell proliferation and migration [21, 22].

circRNAs have different modes of intracellular regulatory action; miRNAs can be regulated by sponging, constituting a circRNA-miRNA-mRNA interplay network and forming a ceRNA mechanism [23]. circRNAs can regulate the expression of downstream target genes via ceRNA mechanisms [24]. Using bioinformatics analysis, we identified the binding sites for microRNA-1303 (miR-1303) on hsa_circ_0002980, which may function as a sponge for adsorbing miR-1303. We also predicted the target genes of miR-1303 using bioinformatics and found that CADM2 might be targeted by miR-1303. Furthermore, CADM2 has been shown to prevent the progression of cancers, such as retinoblastoma [25], breast cancer [20], glioma [22].

Based on the above analysis, we included LC in our study and adopted molecular and cellular functional assays to investigate the role of hsa_circ_0002980 in LC cells. We also verified the regulatory effect of hsa_circ_0002980 on miR-1303 and CADM2 expression. In summary, we identified new targets and approaches for LC therapy.

## MATERIALS AND METHODS

### Tissue samples

Twenty LC tissues and their adjacent tumor tissues (>5 cm from the tumor margin) were obtained from patients diagnosed with LC at Zhongshan Hospital. All patients underwent surgery and signed an informed consent form. The participants had complete clinical data and were not treated with chemotherapy or radiotherapy prior to surgery. Fresh specimens were stored in liquid nitrogen for rapid freezing immediately after resection. This study was approved by the Institutional Review Board of Zhongshan Hospital (Registration No. xmzsyyky (201812)).

### Cell lines

Human liver cells (WRL68) and four LC cell lines (HepG2, Huh7, Li 7 and BEL 7404) were purchased from the National Collection of Authenticated Cell Cultures (NCACC, China). WRL68 cells were incubated in Eagle’s Minimal Essential Medium (EMEM, Mediatech); HepG2 cells were hatched in MEM (Gibco, USA); Huh7 cells were grown in DMEM (Cat. No. P04-04510, Aidenbach; Germany); Li 7 and BEL 7404 cells were cultured in RPMI-1640 medium (Cat. No. 11875). All media contained 10% fetal bovine serum (Gibco, USA) and 1% penicillin/streptomycin (Cat. No. P0781, Sigma, USA), and all cells were grown at 37°C in a 5% CO_2_ incubator. Trypsin digestion was performed, and cells in the logarithmic growth phase were used for experiments.

### Cell transfection

The empty vector, circ_0002980 overexpression plasmid, and CADM2 overexpression plasmid were provided by Integrated Biotech Solutions (Shanghai, China). miR-1303 mimics and a negative control (NC) were designed and synthesized by GenePharm (Shanghai, China). The sequence of miR-1303 mimic was 5′-UUUAGAGACGGGGUCUUGCUCU-3′; the sequence of mimic NC was 5′-UUCUCCGAACGUGUCACGU-3′. HepG2 and Huh7 cells (density, approximately 50%) were transfected using Lipofectamine 3000 (Invitrogen, USA). Cells were individually transfected with circ_0002980 overexpression plasmids, co-transfected with circ_0002980 overexpression plasmids and miR-1303 mimics, or co-transfected with CADM2 overexpression plasmids and miR-1303 mimics using Lipofectamine 3000 (Invitrogen) based on the manufacturer’s instructions. After transfection, the cells were cultured in incubators for 48 h and collected for experimental detection.

### Quantitative real-time PCR (RT-qPCR)

Total RNA was extracted from each group of cells or milled tissue samples using TRIzol reagent. After testing RNA concentration and purity, RNA was reverse transcribed to cDNA by PrimeScript™ RT reagent kits (Takara, Dalian, China). The procedure was undertaken at 42°C for 2 min in the first step; 37°C for 15 min, and 85°C for 5 s in the second step. The cDNA obtained by reverse transcription was used as a template, and the SYBR Green PCR kit (Applied Biosystems, USA) was used for RT-qPCR. The relative quantification method was adopted to determine the Ct values of the gene PCR products, and the quantification results were analyzed through the 2^−ΔΔCt^ method.

### circRNA microarray sequencing

The extracted RNA was purified using a RNeasy MinElute Cleanup Kit (Qiagen, Netherlands). Specific libraries were constructed using Illumina’s VAHTS™ Total RNA-seq (H/M/R) (Vazyme, China) and sequenced using HiSeq™ 2500 (Illumina, USA). Intergroup differences were analyzed using the edgeR software package (http://www.r-project.org/, 3.12.1). Differentially expressed circRNAs are represented by a heat map.

### Clone formation assay

Transfected HepG2 and Huh7 cells (200 cells/well) were uniformly inoculated into 6-well plates. After 10 days of conventional culture, 4% paraformaldehyde was used to fix the cells for 20 min and 0. 1% crystalline violet was applied to stain the cells for 5 min. Subsequently, photographs were taken for statistical analysis.

### Flow cytometer

Cells (3 × 10^5^ cells/well) were seeded in 6-well plates and cultured to the normal growth stage. The EdU was diluted with the medium at a ratio of 1:1000 to prepare 50 μM EdU solution. A control group lacking the EdU medium was used. After transfection, the cell medium was replaced with 1 mL EdU solution and incubated for 2 h. Subsequently, the collected cells were centrifuged and resuspended in PBS, they were treated with 300 μL of 1× Apollo solution, and kept out of light for 10 min. After washing, the number of EdU-positive cells was determined using flow cytometry (BD FACSCalibur).

### Transwell

For migration, transfected HepG2 and Huh7 cells were resuspended in serum-free media. Thereafter, 5 × 10^4^ cells were positioned in the upper chamber of each Transwell (8 μm, Corning, USA) and 500 μL of culture medium with 10% FBS was placed in the lower chamber. After 24 h of routine incubation, the culture medium was discarded and the cells were washed three times and fixed in 4% paraformaldehyde for 20 min. The liquid in the upper chamber was removed using cotton swabs, and the cells were stained with 1% crystal violet for 10 min. After washing, the cells were photographed under a microscope in five random fields. For invasion assay, pre-cooled Matrigel (Cat. No. 356234, EMD Millipore, USA) was diluted in a serum-free medium, at a ratio of 1:8. Subsequently, 80 μL of the diluted Matrigel was spread on the upper chamber of the Transwell to evenly coat the bottom of the chamber at 37°C for 60 min to form the gel. The remaining steps were identical to those used in the migration experiment.

### Western blot

Following transfection for 48 h, each group of cells was collected and 100 μL of protein lysate (PMSF preparation) was added, then bathed in ice for 30 min. After centrifugation (4°C, 12000 r/min, 10 min), the supernatant (2 μL) was applied to confirm the protein concentration by BCA kit (Beyotime, China), and the remaining supernatant was stored in the fridge (−20°C). Fifty micrograms of protein were mixed with 5× loading buffer and boiled in a water bath for 10 min. The denatured samples were subjected to sodium dodecyl sulfate-polyacrylamide gel electrophoresis (voltage 80V for 30 min; 110V for 40 min). After completion of electrophoresis, the protein gel was transferred to a 0.22 μm PVDF membrane by semi-dry transfer instrument (Bio-Rad, USA). Following transfer, the membranes were blocked with 5% skim milk powder for 2 h. Thereafter, the membranes were incubated in the diluted primary antibodies (Abcam, UK) at 4°C with overnight shaking. After washing 3 times with TBST solution, the secondary antibody (Abcam) was used for incubation. After 2 h, the membrane was washed three times with TBST before exposure. After processing with Pierce™ ECL substrate (Thermo Fisher Scientific), signals were obtained using an ECL system (Thermo Fisher Scientific) and the data was analyzed by Image J.

### Dual luciferase reporter assay

RegRNA 2.0 was used to predict the potential binding genes of circ_0002980 and miR-1303. Moreover, TargetScan (http://targetscan.org) was used to predict the binding of miR-1303 to CADM2. circ_0002980 and CADM2 3′UTR were first amplified by PCR, respectively. Next, circ_0002980 and CADM2 wild-type (WT) vectors were constructed, and circ_0002980 and CADM2 mutants (Mut) were generated using point mutation kits. Huh7 cells (1 × 10^5^ cells/well) in a 6-well plate were transfected with recombinant plasmids and miR-1303 mimics using Lipofectamine 3000 (Invitrogen). Luciferase activity was measured using a detection kit (Promega Corporation, USA).

### Statistical analysis

Data are denoted as mean ± standard deviation (SD) from at least three independent experiments. Statistical analysis was performed using SPSS software (version 23.0; SPSS Inc., USA); GraphPadPrism 8.0 (USA) was utilized for statistical graph production. The comparison between multiple groups was confirmed through one-way ANOVA, and the least significant difference (LSD) *t*-test was applied for the comparison between two groups. Statistical significance was set at *P* < 0.05.

## RESULTS

### hsa_circ_0002980 was lowly expressed in LC

**Figure 1:**
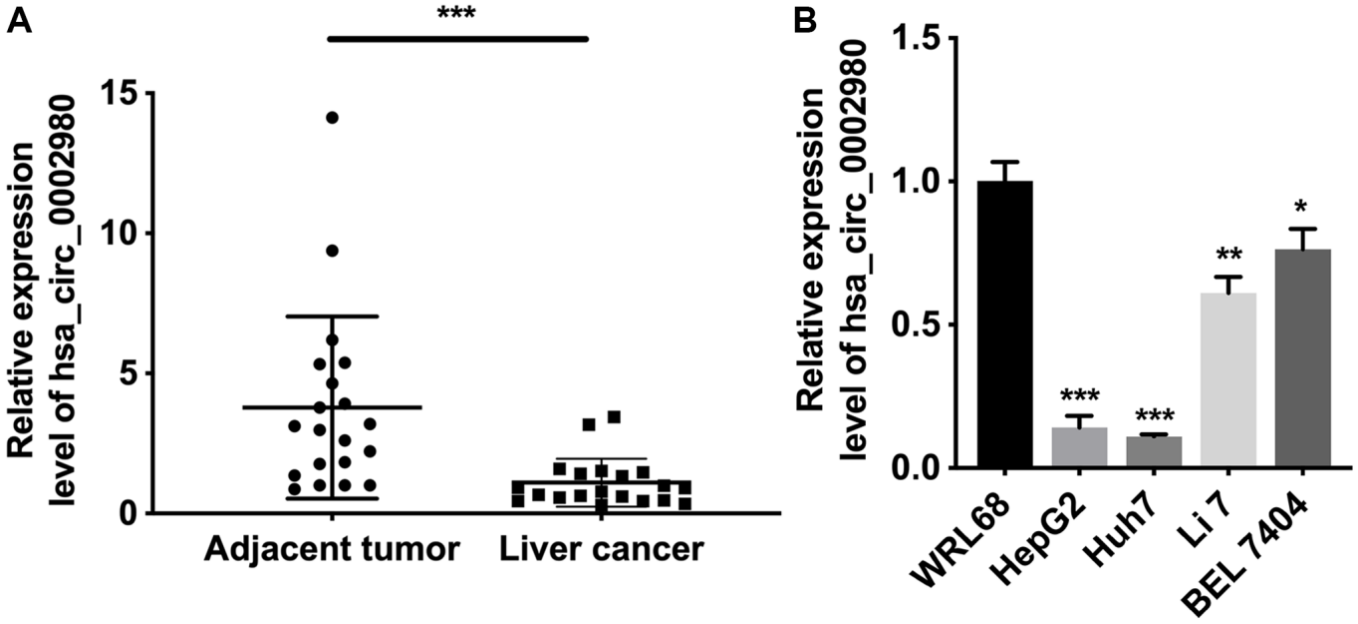
**hsa_circ_0002980 was lowly expressed in LC.** (**A**) RT-qPCR analysis of hsa_circ_0002980 in LC tissues (*n* = 20) and adjacent tumor tissues (*n* = 20). (**B**) The level of hsa_circ_0002980 was identified via RT-qPCR in human liver cells (WRL68) and four LC cells (HepG2, Huh7, Li 7 and BEL 7404). ^*^P < 0.05; ^**^P < 0.01; ^***^P < 0.001.

We first applied circRNA microarray sequencing to screen differentially expressed circRNAs between LC tissues and adjacent tumor tissues, which are displayed using a heap map (Supplementary Figure 1). Based on the screening results, we selected hsa_circ_0002980, which showed large differences in downregulation, as the research target. We used RT-qPCR to test hsa_circ_0002980 expression in the 20 LC and adjacent tumor tissues. The results indicated that hsa_circ_0002980 was expressed in both LC and adjacent tissues, and that the expression of hsa_circ_0002980 in LC tissues was lower than that in the adjacent tissues (Figure 1A). Simultaneously, we determined the expression of hsa_circ_0002980 in LC. As shown in Figure 1B, hsa_circ_0002980 levels were significantly reduced in LC cells (HepG2, Huh7, Li 7 and BEL 7404) relative to those in human liver cells (WRL68), especially in HepG2 and Huh7 cells. In summary, these data revealed that hsa_circ_0002980 is downregulated in LC, indicating that circ_0002980 expression is relevant to the occurrence of LC and may participate in the control of its carcinogenesis.

### Overexpression of circ_0002980 memorably suppressed the proliferation of LC cells

**Figure 2:**
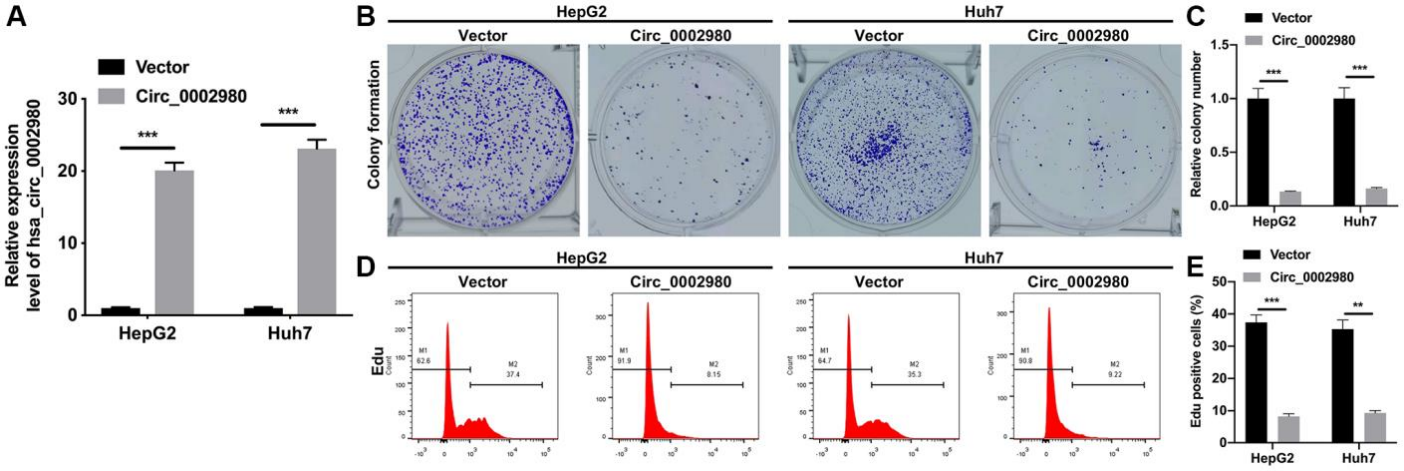
**Overexpression of circ_0002980 memorably suppressed the proliferation of LC cells.** (**A**) HepG2 and Huh7 cells were overexpressed by circ_0002980 through transfection, and the overexpression effect of circ_0002980 was verified with RT-qPCR. (**B**) Clone formation assay was applied to evaluate the change of clone-forming ability in circ_0002980-overexpressed HepG2 and Huh7 cells. (**C**) Quantitative analysis of relative colony number. (**D**) After circ_0002980 overexpression, cell proliferative potential was monitored using flow cytometer with Edu dye. (**E**) Edu positive cells were quantified. ^**^P < 0.01; ^***^P < 0.001.

To explore the possible roles of circ_0002980 in LC, we overexpressed circ_0002980 in HepG2 and Huh7 cells and observed its impact on the proliferation of LC cells through relevant experiments. RT-qPCR results indicated that circ_0002980 levels were markedly increased in the circ_0002980 transfection group compared with the vector group. This revealed the effective transfection of the circ_0002980 overexpression plasmid in HepG2 and Huh7 cells (Figure 2A). Clone formation data showed that the colony number was significantly diminished in the circ_0002980 overexpression group compared with that in the vector group (Figure 2B, 2C). Flow cytometry data showed that the cell proliferative potential was also reduced in the circ_0002980 overexpression group compared with that in the vector group (Figure 2D, 2E). Overall, we found that circ_0002980 overexpression inhibited LC cell proliferation.

### circ_0002980 overexpression markedly weakened the migration, invasion, and EMT of LC cells

**Figure 3:**
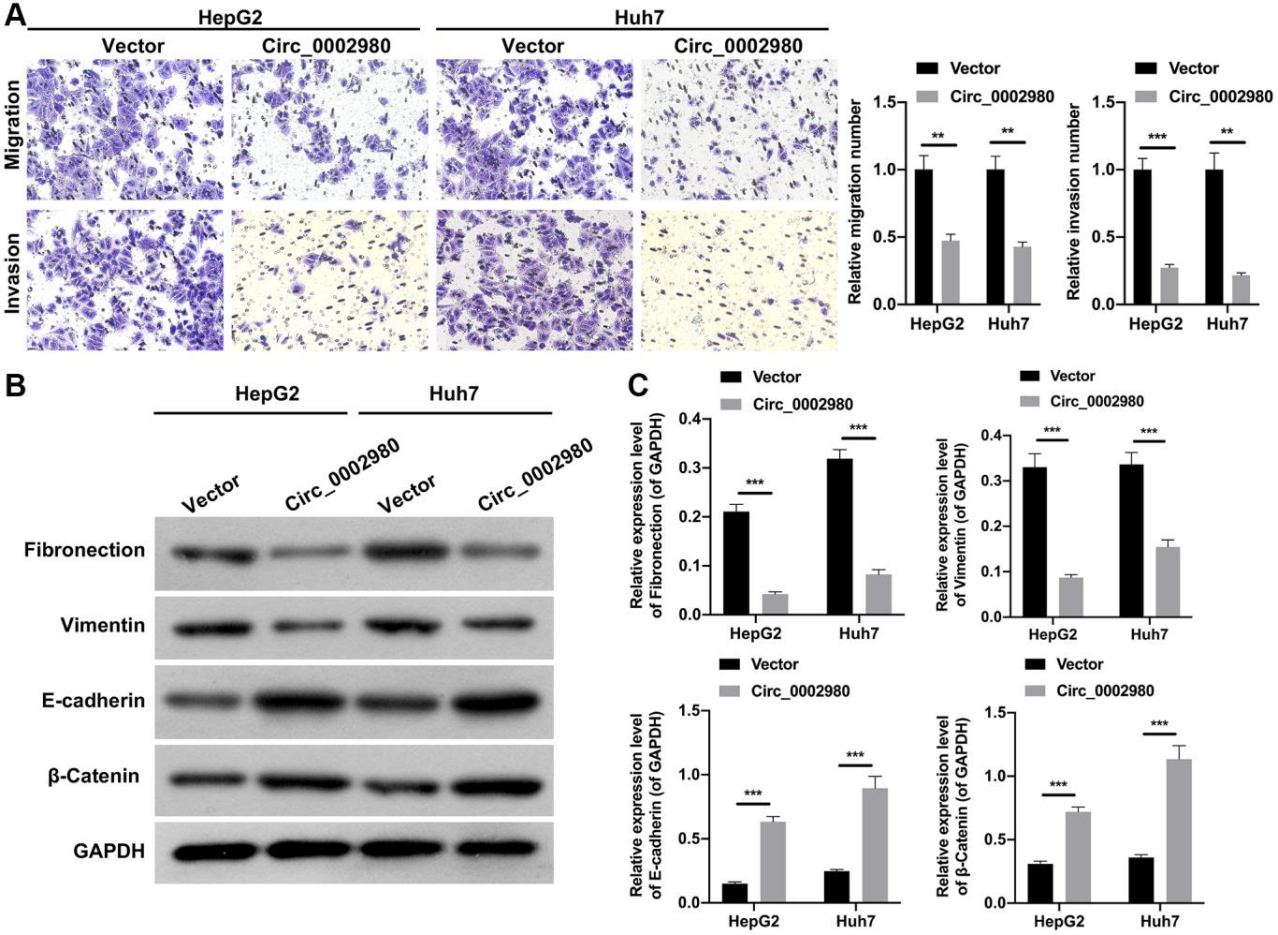
**circ_0002980 overexpression markedly weakened the migration, invasion, and EMT of LC cells.** (**A**) We adopted Transwell assays to identify the impact of circ_0002980 overexpression on the migration and invasion capacities of LC cells. Magnification, 200×. (**B**) The levels of EMT-related proteins (Fibronectin, Vimentin, E-cadherin, and β-Catenin) were analyzed by applying Western blot in circ_0002980-overexpressed LC cells. (**C**) Western blotting results were quantitatively analyzed. ^**^P < 0.01; ^***^P < 0.001.

We also studied the effect of circ_0002980 overexpression on LC cell metastasis and EMT. Transwell data showed that relative to the vector group, the number of migrating and invading cells was markedly decreased after circ_0002980 overexpression in HepG2 and Huh7 cells (Figure 3A). Moreover, Western blotting data denoted that circ_0002980 overexpression could notably lower Fibronectin and Vimentin expression, and heighten E-cadherin and β-Catenin expression in LC cells. Indicating that circ_0002980 overexpression could inhibit EMT progression of LC cells (Figure 3B, 3C). Thus, we confirmed that circ_0002980 overexpression prevented metastasis and EMT development.

### circ_0002980 overexpression restrained LC cell proliferation through miR-1303

**Figure 4:**
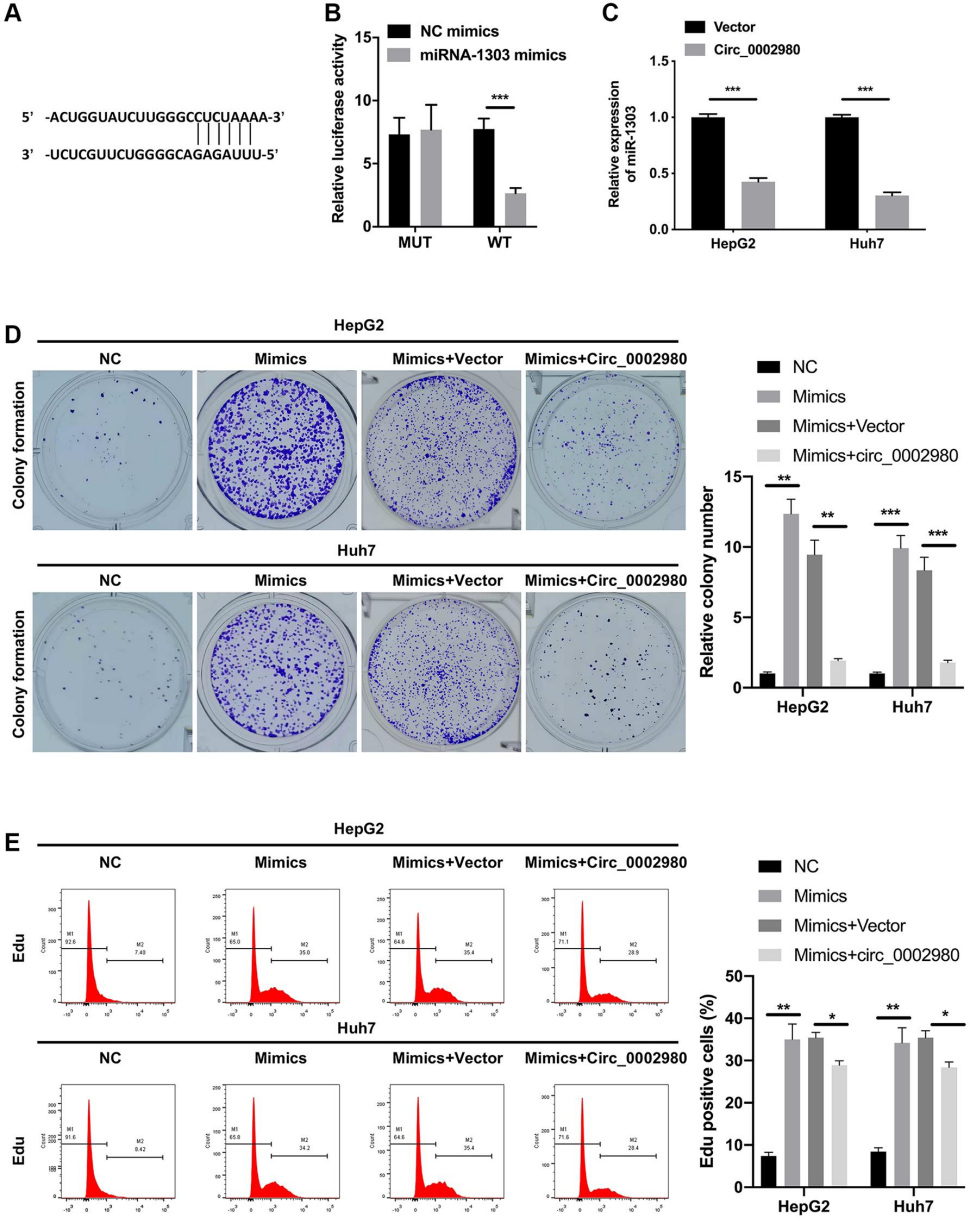
**circ_0002980 overexpression signally restrained LC cell proliferation through miR-1303.** (**A**) The binding sites of circ_0002980 and miR-1303 were predicted using RegRNA software. (**B**) We applied dual luciferase reporter assays to analyze the changes in luciferase activity of WT- or Mut-circ_0002980. (**C**) RT-qPCR was utilized to identify miR-1303 expression after circ_0002980 overexpression. (**D**) HepG2 and Huh7 cells were then co-transfected with miR-1303 mimics and circ_0002980 overexpression plasmids, and the clone-forming ability was credited via clone formation assay. (**E**) After Edu dyeing, flow cytometer was utilized to confirm the proliferative potential in LC cells treated as D. ^*^P < 0.05; ^**^P < 0.01; ^***^P < 0.001.

We also utilized bioinformatics software (RegRNA) to predict the miRNAs possibly regulated by circ_0002980. Further, miR-1303 was identified by screening. We identified a binding site between circ_0002980 and miR-1303 (Figure 4A). We discovered that after miR-1303 overexpression, the luciferase activity of circ_0002980 was markedly decreased when the plasmid carried WT-circ_0002980 3′-UTR. Meanwhile there was no change in the luciferase activity of circ_0002980, when the plasmid carried Mut-circ_0002980 3′-UTR, suggesting a targeting relationship between miR-1303 and circ_0002980 (Figure 4B). We also found that circ_0002980 overexpression significantly downregulated miR-1303 expression in LC cells (Figure 4C). To confirm the influence of miR-1303 and circ_0002980 on the malignant behavior of LC cells, HepG2 and Huh7 cells were co-transfected with miR-1303 mimics and circ_0002980 overexpression plasmids. Clone formation results indicated that the upregulation of miR-1303 dramatically elevated the clone-forming ability of LC cells, and this elevation could also be significantly decreased by circ_0002980 overexpression (Figure 4D). Flow cytometry results showed that the upregulation of miR-1303 notably increased the proliferative potential of LC cells, while the increase in miR-1303-mediated proliferation was reversed by circ_0002980 overexpression (Figure 4E). Therefore, we further demonstrated that the inhibitory effect of circ_0002980 on the proliferation of LC cells can be realized by downregulating miR-1303 expression.

### circ_0002980 overexpression dramatically prevented the migration, invasion, and EMT of LC cells through miR-1303

**Figure 5:**
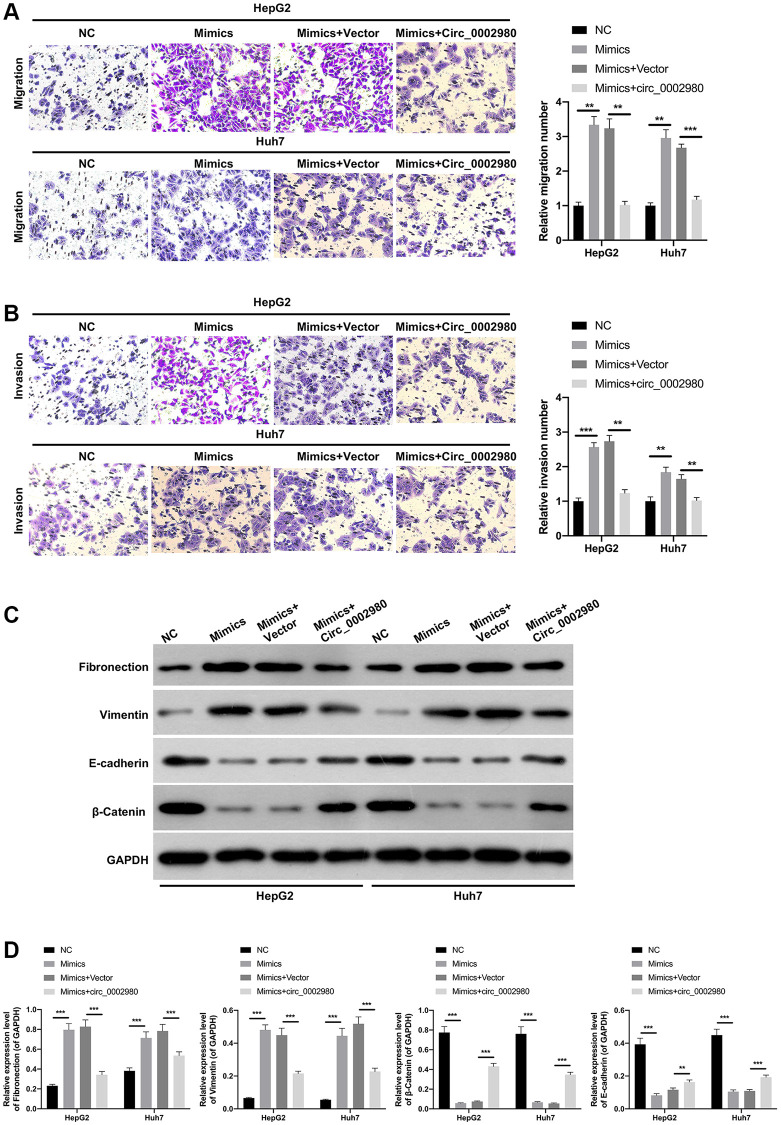
**circ_0002980 overexpression dramatically prevented the migration, invasion, and EMT of LC cells through miR-1303.** miR-1303 mimics and circ_0002980 overexpression plasmids were transfected into HepG2 and Huh7 cells, respectively. (**A**) After transfection, Transwell assay was performed to certify the change in cell migration ability. Magnification, 200×. (**B**) The invasion capacity of the processed cells was observed by Transwell assay. Magnification, 200×. (**C**) The levels of EMT-related proteins in the transfected HepG2 and Huh7 cells were monitored using Western blot. (**D**) The expression levels of four proteins were calculated in line with the grey values. ^**^P < 0.01; ^***^P < 0.001.

We investigated the effects of miR-1303 and circ_0002980 on LC metastasis and EMT. Transwell results displayed that the migrated and invaded cells were notably increased in miR-1303 mimics group compared with that in the NC group, the induction of migration and invasion by miR-1303, while this induction also could be dramatically attenuated by circ_0002980 overexpression in LC cells (Figure 5A, 5B). Meanwhile, Western blot data exhibited that relative to the NC group, Fibronectin and Vimentin expression was memorably increased, E-cadherin and β-Catenin expression was prominently decreased in miR-1303 mimics group, while the expression changes of these four proteins mediated by miR-1303 also could be dramatically reversed by circ_0002980 overexpression in LC cells (Figure 5C, 5D). These results suggest that circ_0002980 reduces metastasis and EMT of LC cells by downregulating miR-1303 expression.

### CADM2 was a target gene of miR-1303

**Figure 6:**
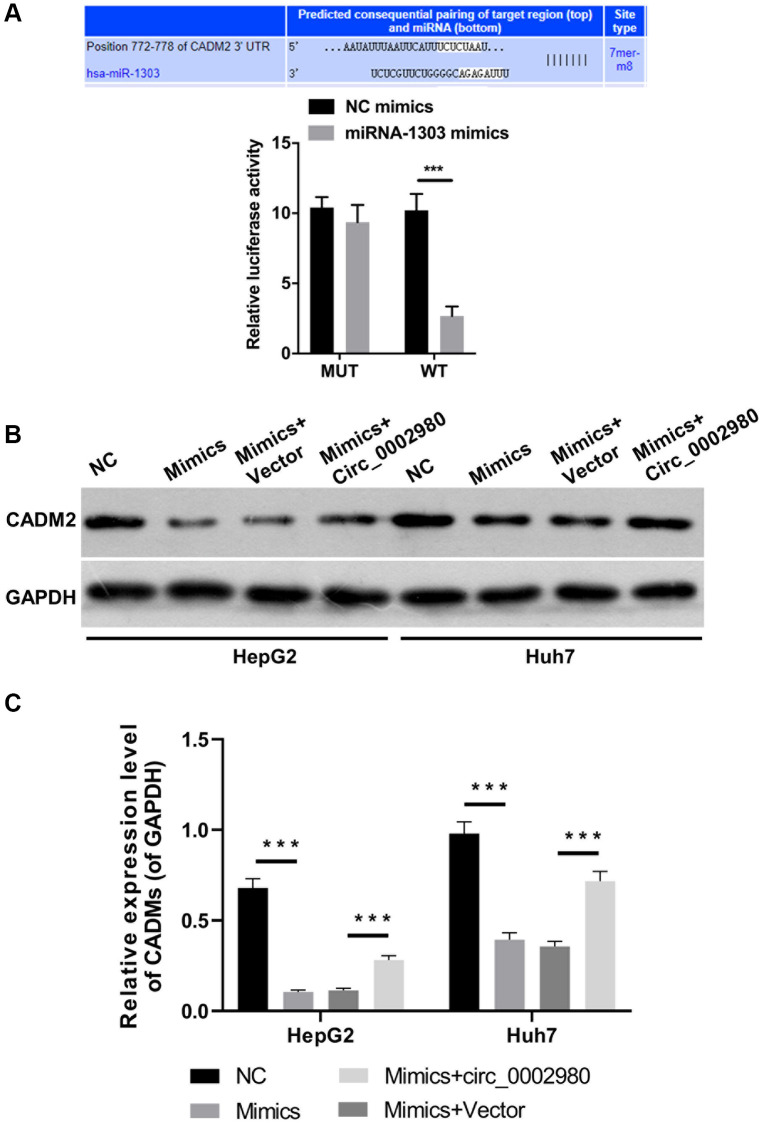
**CADM2 was a target gene of miR-1303.** (**A**) TargetScan analysis was applied to predict the binding site between miR-1303 and CADM2, and the targeting effect of miR-1303 to CADM2 was confirmed using dual luciferase reporter assay. (**B**) Western blotting analysis of CADM2 expression in HepG2 and Huh7 cells, which were administrated with miR-1303 mimics and circ_0002980 overexpression plasmids. (**C**) Quantitative analysis of CADM2 protein expression.

Target genes that may be regulated by miR-1303 were further analyzed using bioinformatics (TargetScan). Through analysis and screening, we determined that CADM2 is likely to be a target gene of miR-1303. The dual luciferase reporter data indicated that overexpression of miR-1303 markedly reduced the luciferase activity of WT-CADM2 but had no effect on the luciferase activity of Mut-CADM2, proving a targeting relationship between miR-1303 and CADM2 (Figure 6A). Western blotting data showed that an increase in miR-1303 significantly downregulated CADM2, whereas circ_0002980 overexpression upregulated CADM2 expression mediated by miR-1303 in LC cells (Figure 6B, 6C).

### miR-1303 partially reversed CADM2-mediated prevention in proliferation, migration and invasion of LC cells

**Figure 7:**
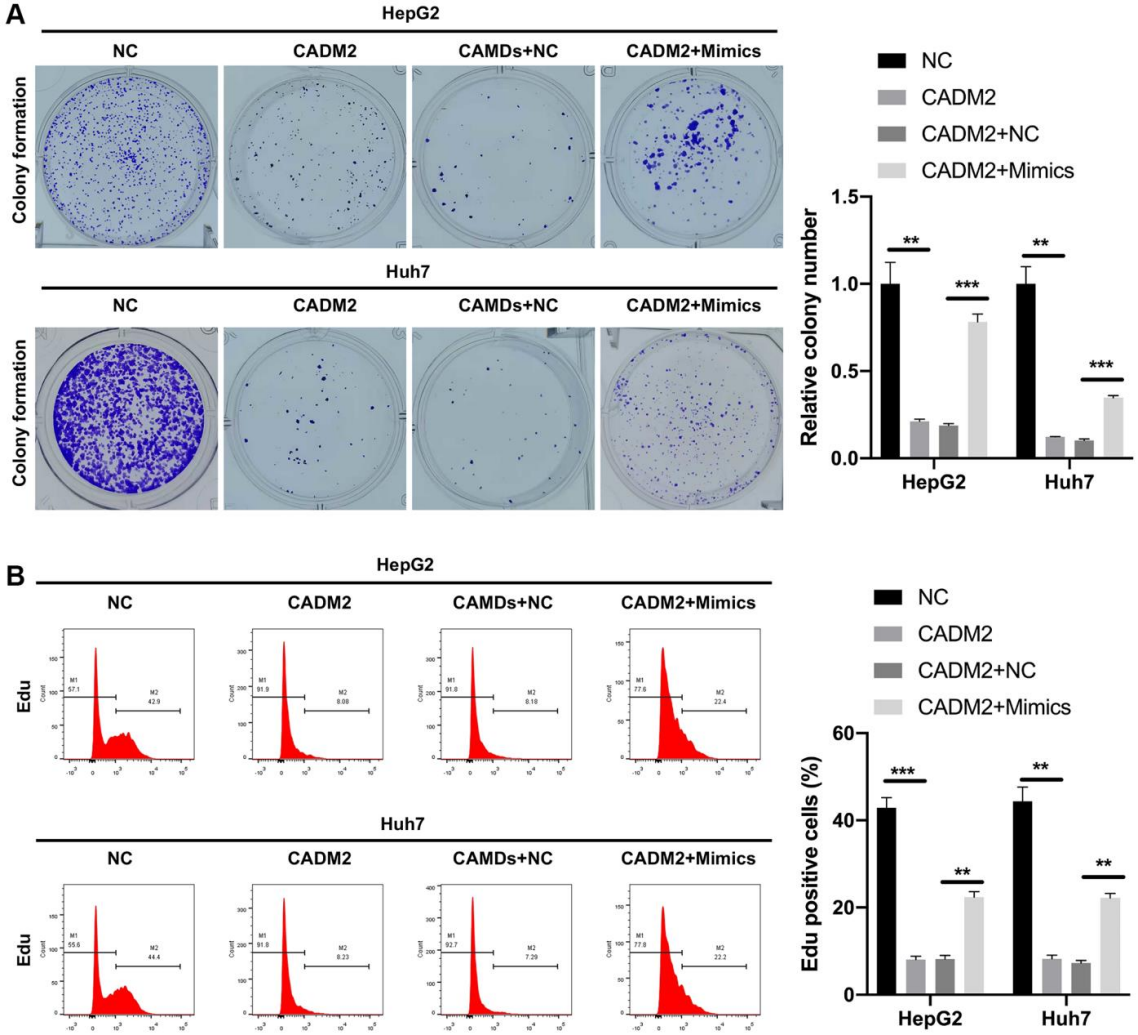
**miR-1303 partially reversed CADM2-mediated prevention in proliferation of LC cells.** (**A**) Clone formation assay exhibited the change in clone-forming ability in HepG2 and Huh7 cells, which were cotransfected with CADM2 overexpression plasmids and miR-1303 mimics. (**B**) Flow cytometer was adopted to verify the change of cell proliferation in each group. ^*^P < 0.05; ^**^P < 0.01; ^***^P < 0.001.

**Figure 8:**
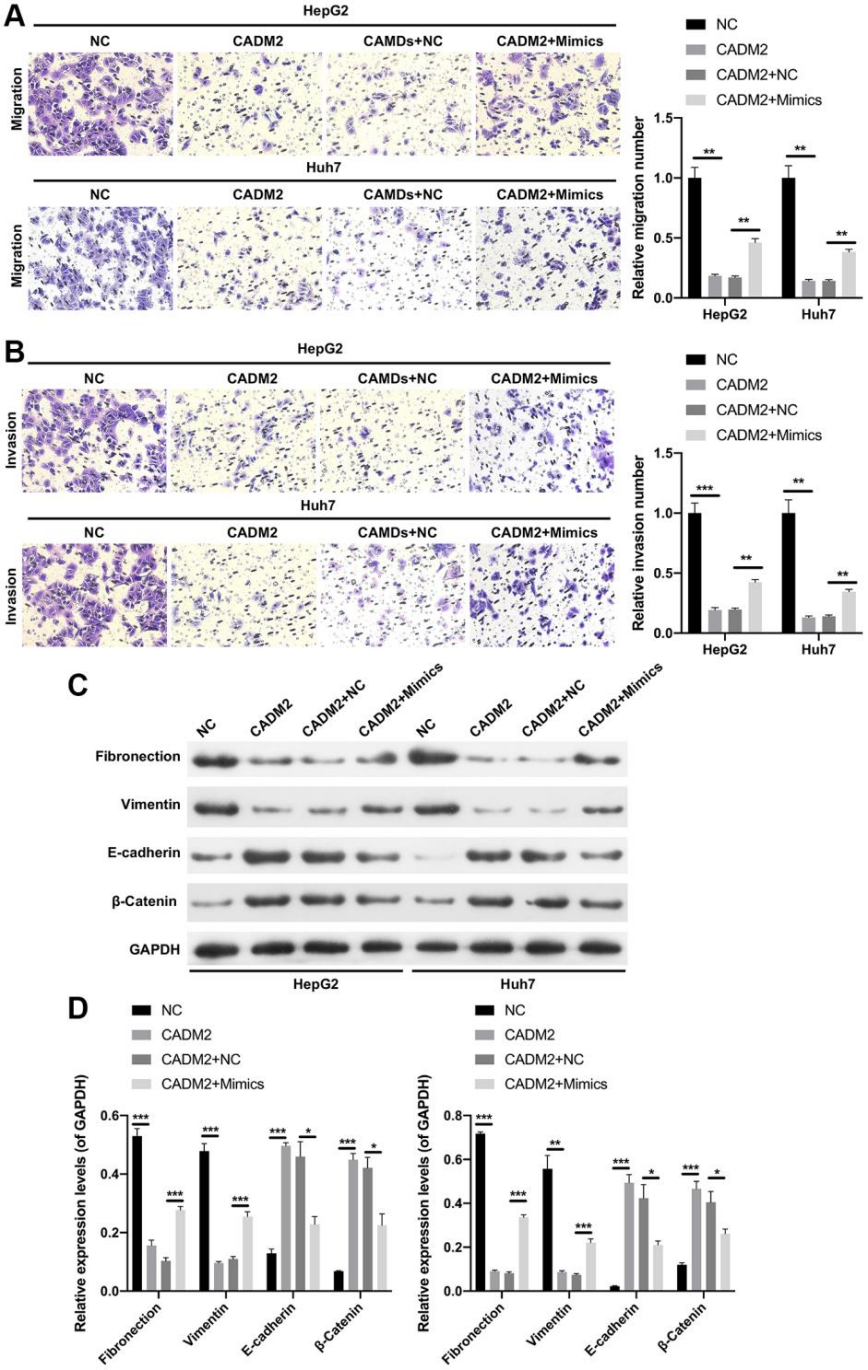
**miR-1303 attenuated CADM2-mediated inhibition in migration and invasion of LC cells.** HepG2 and Huh7 cells were transfected with miR-1303 mimics and circ_0002980 overexpression plasmids. (**A**) The ability of cell migration was viewed via Transwell assay in each group. Magnification, 200×. (**B**) Cell invasion was tested through Transwell in each group. Magnification, 200×. (**C**) Western blotting analysis of EMT-related proteins. (**D**) Quantitative analysis of proteins. ^*^P < 0.05; ^**^P < 0.01; ^***^P < 0.001.

Functionally, CADM2 overexpression markedly decreased the clone-forming ability of LC cells, whereas the decrease in colony count mediated by CADM2 was prominently reversed by miR-1303 overexpression (Figure 7A). In addition, our data showed that CADM2 overexpression significantly reduced the number of Edu-positive cells. Meanwhile, the reduction in the proliferation ability mediated by CADM2 could also be reversed by miR-1303 overexpression in LC cells (Figure 7B). Moreover, Transwell assays indicated that the inhibition of migration and invasion mediated by CADM2 overexpression could also be significantly weakened by miR-1303 overexpression in LC cells (Figure 8A, 8B). Meanwhile, we discovered that downregulation of Fibronectin and Vimentin, and upregulation of E-cadherin and β-Catenin mediated by CADM2 overexpression could also be prominently restored by miR-1303 overexpression in LC cells (Figure 8C, 8D). We further verified that CADM2, as a target gene, was crucial for blocking the progression of LC mediated by miR-1303.

## DISCUSSION

The American Cancer Society has stated that the incidence and mortality rates of malignancies other than LC have declined worldwide [6]. Evidence suggests that LC may be caused by oncogene activation, hepatitis B virus (HBV) infection, or inactivation of oncogenes. However, the mechanisms underlying LC progression remain unclear [26]. Therefore, further investigation into the mechanisms underlying the development of LC is crucial for its clinical management. circRNAs are a class of first- and tail-covalently bound circular RNAs that regulate the malignant behavior of LC [15]. Moreover, aberrantly expressed circRNAs can be used to distinguish LC from healthy populations, predict patient prognosis, and correlate with clinical factors, including microvascular infiltration, metastasis, and methotrexate levels [13]. Therefore, circRNAs may serve as novel therapeutic targets for LC. However, the detailed functions and critical mechanisms of the circRNAs in LC remain largely unknown. In this study, we screened circ_0002980 using circRNA microarray sequencing. We demonstrated that circ_0002980 was expressed at low levels in LC and that circ_0002980 overexpression prevented the proliferation of LC cells.

EMT is one of the mechanisms regulating the metastasis of tumor cells, which can cause weakened adhesion and increased motility of tumor cells, resulting in greater invasive capacity [27]. The EMT process is often accompanied by changes in the cytoskeleton and related phenotypic genes (downregulation of E-Cadherin and β-Catenin, and upregulation of Fibronectin and Vimentin) [28]. Recent studies have shown that aberrant expression of circRNAs in certain tumors is involved in the EMT process, which in turn regulates the malignant behavior of tumors [29, 30]. Therefore, the present study was conducted to further investigate the effects of circ_0002980 on the EMT and invasion of LC cells. The results revealed that circ_0002980 overexpression inhibited the EMT, migration, and invasion of LC cells. Therefore, this study provides preliminary evidence for the potential function of circ_0002980 in LC cells. However, the specific regulatory mechanism of hsa-circ_0002980 in LC requires further exploration.

In cancer, a key mechanism by which circRNAs exert their function is by binding to tumor-associated miRNAs and suppressing their function [31]. MiRNAs, a group of single-stranded RNAs, play a crucial role in tumor progression, especially in LC [32, 33]. Multiple studies have demonstrated that miRNAs can influence LC progression by regulating cell proliferation, apoptosis, metastasis and immune escape [33, 34]. Wei et al. found that circ_0002980 promotes dermal papilla cell apoptosis through the miR-3180-5p/BAX signaling axis. This axis mediates androgenic alopecia (AGA), providing novel targets and ideas for AGA [35]. In this study, miR-1303 was identified as a possible target miRNA of circ_0002980 using bioinformatics prediction. Our results further show that circ_0002980 overexpression prevents LC through miR-1303. Therefore, circ_0002980 may exert its cancer-suppressive effect by suppressing miR-1303, which has oncogenic effects. It has been reported that miR-1303 is indeed carcinogenic in various cancers [36, 37].

In addition, circRNAs regulate target gene expression by acting as ceRNAs. We adopted TargetScan prediction to identify CADM2 as an miR-1303 target. Furthermore, this study further confirmed the targeted relationship between CADM2 3′-UTR and miR-1303. Moreover, our study indicated that miR-1303 induces the proliferation and metastasis of LC cells through CADM2.

In summary, this study showed that circ_0002980 was expressed at low levels in LC, and that overexpression of circ_0002980 prevented the proliferation and invasion of LC cells. In addition, circ_0002980 may play an oncogenic role by suppressing miR-1303 in order to upregulate CADM2 expression. Therefore, the circ_0002980/miR-1303/CADM2 axis may be an effective clinical therapeutic target in LC. In future studies, we will verify the role of the circ_0002980/miR-1303/CADM2 axis in LC using a large number of animal models. In addition, we plan to expand the clinical tissue and serum samples of patients with LC to further verify the expression of circ_0002980, miR-1303, and CADM2 in LC as well as their prognostic value. Through this study, and future investigations, we aim to develop effective diagnostic markers and therapeutic targets for LC.
